# Key molecular alterations in endothelial cells in human glioblastoma uncovered through single-cell RNA sequencing

**DOI:** 10.1172/jci.insight.150861

**Published:** 2021-08-09

**Authors:** Yuan Xie, Liqun He, Roberta Lugano, Yanyu Zhang, Haiyan Cao, Qiyuan He, Min Chao, Boxuan Liu, Qingze Cao, Jianhao Wang, Yang Jiao, Yaqin Hu, Liying Han, Yong Zhang, Hua Huang, Lene Uhrbom, Christer Betsholtz, Liang Wang, Anna Dimberg, Lei Zhang

**Affiliations:** 1 Key Laboratory of Ministry of Education for Medicinal Plant Resource and Natural Pharmaceutical Chemistry, National Engineering Laboratory for Resource Developing of Endangered Chinese Crude Drugs in Northwest of China, China-Sweden International Joint Research Center for Brain Diseases, College of Life Sciences, Shaanxi Normal University, Xi’an, China.; 2 Department of Immunology, Genetics and Pathology, Science for Life Laboratory, Uppsala University, Rudbeck Laboratory, Uppsala, Sweden.; 3 Department of Neurosurgery, Tianjin Medical University General Hospital, Tianjin Neurological Institute, Key Laboratory of Post-Neuro-injury Neuro-Repair and Regeneration in Central Nervous System, Ministry of Education, Tianjin City, Tianjin, China.; 4 Department of Neurosurgery, Tangdu Hospital of the Fourth Military Medical University, Xi’an, China.; 5 Precision Medicine Center, The Second People’s Hospital of Huaihua, Huaihua, China.; 6 Genergy Bio-technology (Shanghai) Co. Ltd., Shanghai, China.; 7 Department of Medicine Huddinge (MedH), Karolinska Institutet, Huddinge, Sweden.

**Keywords:** Vascular Biology, Brain cancer, Endothelial cells

## Abstract

Passage of systemically delivered pharmacological agents into the brain is largely blocked by the blood-brain-barrier (BBB), an organotypic specialization of brain endothelial cells (ECs). Tumor vessels in glioblastoma (GBM), the most common malignant brain tumor in humans, are abnormally permeable, but this phenotype is heterogeneous and may differ between the tumor’s center and invasive front. Here, through single-cell RNA sequencing (scRNA-seq) of freshly isolated ECs from human glioblastoma and paired tumor peripheral tissues, we have constructed a molecular atlas of human brain ECs providing unprecedented molecular insight into the heterogeneity of the human BBB and its molecular alteration in glioblastoma. We identified 5 distinct EC phenotypes representing different states of EC activation and BBB impairment, and associated with different anatomical locations within and around the tumor. This unique data resource provides key information for designing rational therapeutic regimens and optimizing drug delivery.

## Graphical abstract

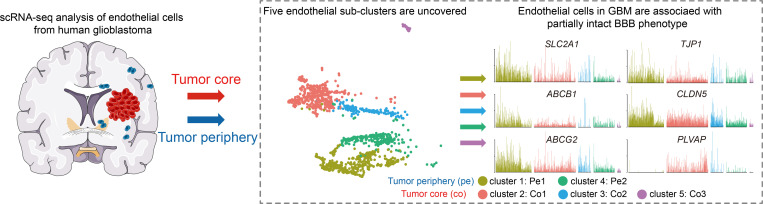

## Introduction

Glioblastoma (GBM) is the most aggressive and lethal type of brain tumor, and the overall survival of the patients has not been improved substantially over the past 30 years (1). The poor prognosis for patients with GBM is at least partially attributed to the extremely limited therapeutic options that are available after surgery and radiotherapy. Despite tremendous basic and clinical research in the GBM field, only 4 drugs have been approved by the FDA for GBM treatment: temozolomide, lomustine, carmustine, and bevacizumab (https://www.cancer.gov/about-cancer/treatment/drugs/brain) (2). However, the effectiveness of these and other antitumor compounds, including the vast majority of low–molecular weight chemotherapeutic drugs, are thwarted by the unique features of the brain’s blood vessels, known as the blood-brain barrier (BBB), which tightly regulate the homeostasis of CNS (3, 4). Failure to cross the brain vessel wall is likely a major contributor to the negative outcome of clinical trials for many blood-borne drugs (2).

Endothelial cells (ECs) are the key cellular component of the BBB. Brain ECs establish continuous complexes of tight and adherens junctions along EC-EC contacts, providing a tight and size-selective barrier. They further express distinct sets of influx and efflux transporters, several of the latter with the ability to bind and limit the brain penetration of a broad variety of small lipophilic xenobiotic compounds and drugs (3). Brain ECs also display very low levels of vesicular transcytosis, further limiting passage of blood-born water-soluble molecules of all sizes (3). Molecular alteration of ECs has been observed in patients and animal models of many brain diseases, including stroke, multiple sclerosis, traumatic brain injury, and GBM (5, 6). By analysis of bulk mRNA isolated from brain ECs, we and others have shown that the abnormal vessels in GBM are associated with a distinct gene signature (7–9). However, the more precise characteristics of endothelial gene expression in GBM, including possible heterogeneity that cannot be resolved using bulk methods, is still poorly understood. Vascular targeting has been tried in order to prune and normalize tumor vessels and, thereby, improve drug delivery in GBM (10–13). However, treatment of patients with bevacizumab to block VEGF signaling did not improve overall survival in unselected patients with GBM, and it also led to a decrease in temozolomide delivery (14).

To understand whether and to what extent ECs in GBM are phenotypically different from control brain ECs at the single-cell level, and to identify potentially novel angiogenic pathways in GBM, we have performed single-cell RNA sequencing (scRNA-seq) on ECs from the human GBM tumor core and tumor peripheral tissue to characterize the heterogeneity of gene expression signatures of different ECs. We have identified 5 EC clusters and found that they were associated with distinct anatomical localizations and molecular phenotypes. Notably, the expression of many BBB-specific transporter mRNAs in GBM ECs was found to be retained to a larger extent than anticipated, and it was heterogeneous rather than uniformly downregulated or absent, together indicating that the BBB phenotype is significantly preserved even among the most abnormal tumor ECs in GBM. To our knowledge, this is the first scRNA-seq–based molecular atlas of the human BBB and its perturbation in GBM. This resource will provide deeper insights into understanding the characteristics of ECs in CNS malignancies, and it will provide information for designing rational therapeutic regimens and optimizing drug delivery.

## Results

### scRNA-seq and cell type identification of GBM and peripheral tissue.

**Figure 1:**
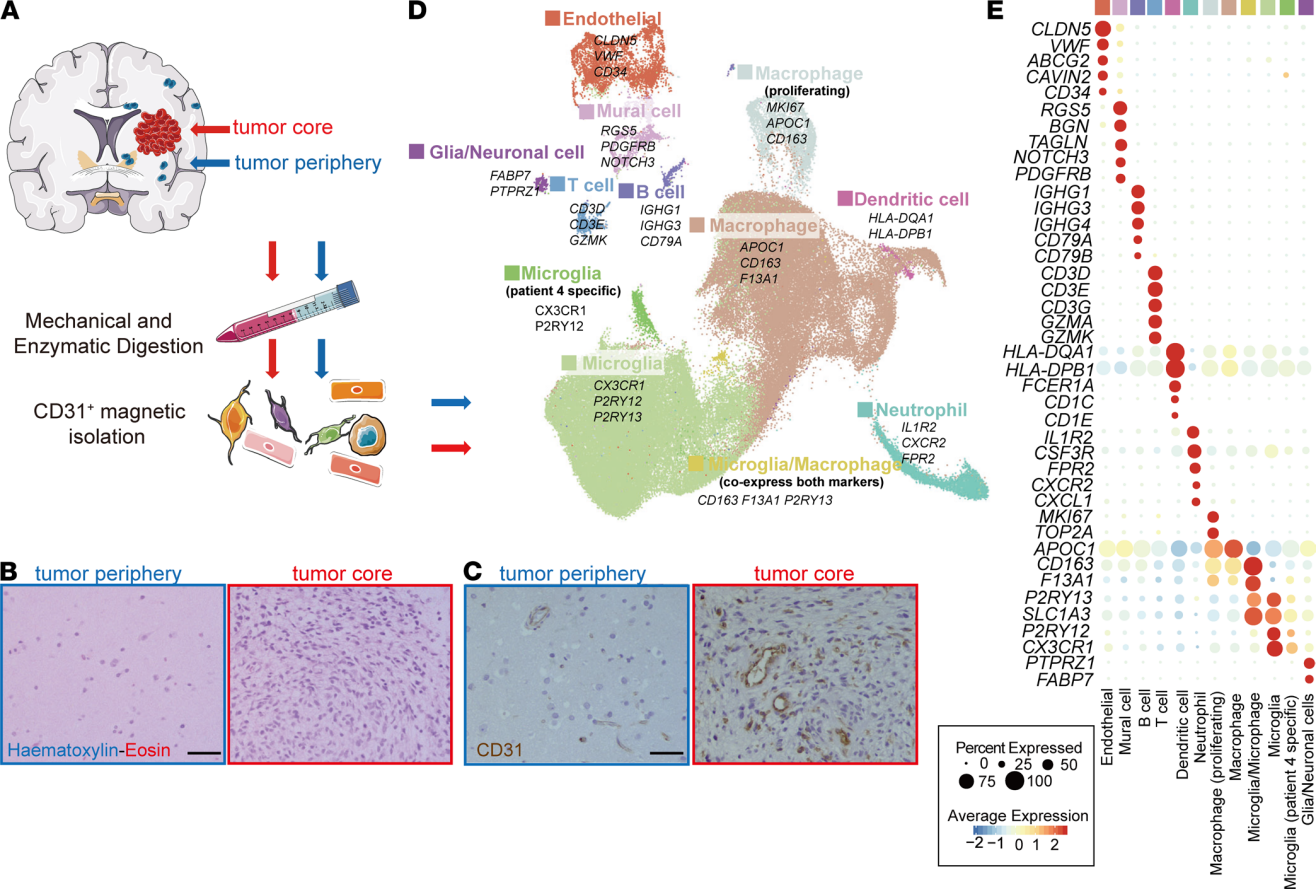
Overview of the CD31-MACS–enriched single cells from GBM and peripheral brain samples. (**A**) Schematic overview of the study design. (**B** and **C**) HE staining (**B**) and IHC staining of CD31 (**C**) in GBM tumor core and tumor peripheral tissue. (**D**) UMAP of transcriptome from CD31-MACS–enriched cells, colored for the 12 clusters. (**E**) Dot plot heatmap of the marker genes in individual clusters. Scale bar: 50 μm.

We isolated cells from 2 distinct regions in each of the surgically removed GBM tissue from 4 patients with GBM: (a) the tumor core and (b) immediately neighboring brain tissue (Figure 1A and Supplemental Table 1; supplemental material available online with this article; https://doi.org/10.1172/jci.insight.150861DS1). All the patients were diagnosed as primary GBM and had not received chemo- or radiotherapy before surgery. Histopathological analysis confirmed a typical GBM picture, including a dense cellularity and abnormal blood vessel profiles within the tumor core, whereas the surrounding tissue showed an overall normal brain histological picture (Figure 1, B and C). GBMs are invariably recurrent in the perioperative region; therefore, we assumed that the surrounding tissues contained tumor cells, albeit at low abundance and without obvious impact in the tissue histology. Importantly, this normality included the vascular morphology, which was reminiscent of that of the normal brain (Figure 1, B and C). Magnetic-activated cell sorting (MACS) was used to generate CD31^+^ cell suspensions, which were subsequently subject to scRNA-seq using a 10X Genomics protocol (Figure 1A). CD31 is an endothelial antigen, which in humans is also expressed abundantly on hematopoietic cells. Due to the limited amount of tissue available (average 1.5 g/sample), additional CD45^–^ selection with the aim of removing leukocytes and enriching for ECs failed to yield sufficient numbers of cells for an optimal 10X Genomics experiment. Thus, CD45^–^ selection was not performed. After quality filtering, we captured 97,584 cells containing, on average, transcript reads from 2224 genes per cell (Supplemental Table 2).

The cells were clustered using the Seurat package (15) and annotated according to expression of canonical cell class markers (Figure 1, D and E, and Supplemental Table 3). In addition to ECs (*CLDN5*, *VWF*, *CD34*) and due to the lack of CD45^–^ selection, other clusters including macrophage (*APOC1*, *CD163*, *F13A1*), microglia (*CX3CR1*, *P2RY12*, *P2RY13*), neutrophils (*IL1R2*, *CXCR2*, *FPR2*), T cells (*CD3D*, *CD3E*, *GZMK*), B cells (*IGHG1*, *IGHG3*, *CD79A*), DCs (*HLA-DQA1*, *HLA-DPB1*), glia/neuronal cells (*FABP7*, *PTPRZ1*), and vascular mural cells (*RGS5*, *PDGFRB*, *NOTCH3*) were also identified in this data set (Figure 1, D and E, and Supplemental Figure 1, A–D).

### Single-cell atlas of EC phenotypes in human brain and GBM.

**Figure 2:**
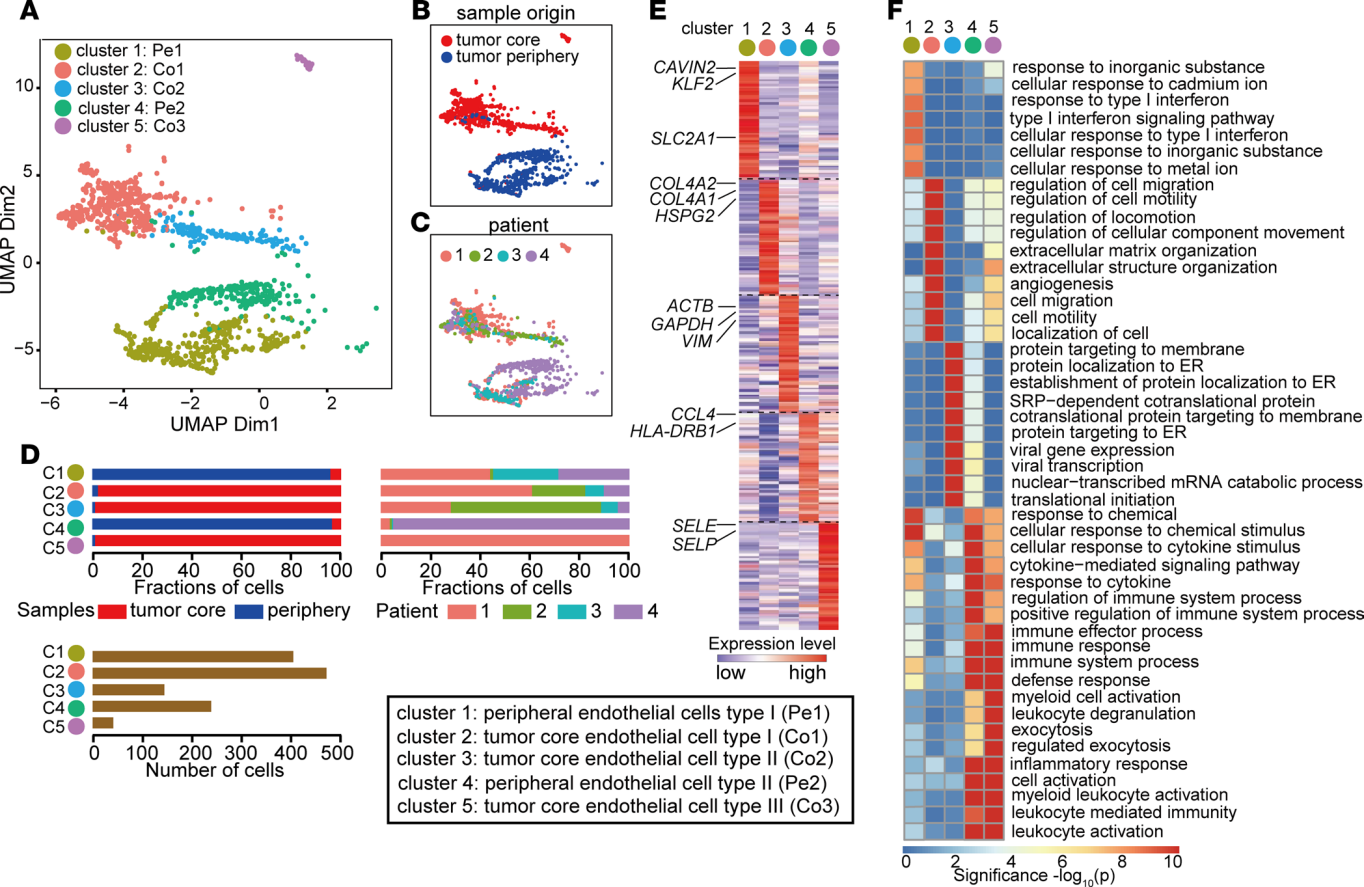
Construction of the EC atlas from GBM and peripheral brain tissue. (**A**–**C**) UMAP of endothelial cells, colored by clusters (**A**) or sample type of origin (**B**) or individual patient (**C**). (**D**) Relative contribution of endothelial cells from sample origin type (left) and individual patient (right). The number of cells in each subclusters (bottom). (**E**) Gene expression levels of top 50 marker genes in different endothelial subclusters. For complete list, see Supplemental Table 4. (**F**) Heatmap showing top 10 enriched GO terms in different endothelial subclusters based on top 50 marker genes.

The ECs were in silico selected based on canonical marker expression (*CLDN5*, *VWF*, *CD34*) and further analyzed. After removal of heterotypic cell doublets, reclustering these revealed 5 EC clusters (clusters 1–5; Figure 2A). Two of the clusters (cluster 1 and cluster 4) were almost entirely originating from brain tissue with nonmalignant morphology surrounding the GBM tumor mass, whereas the remaining 3 clusters (cluster 2, cluster 3, and cluster 5) were largely derived from the tumor core (Figure 2, A–D). Clusters were biologically annotated based on abundance of top-ranking marker genes, together with an enriched gene set signature in each cluster (Figure 2, E and F; Supplemental Table 4; and Supplemental Figure 5).

**Figure 3:**
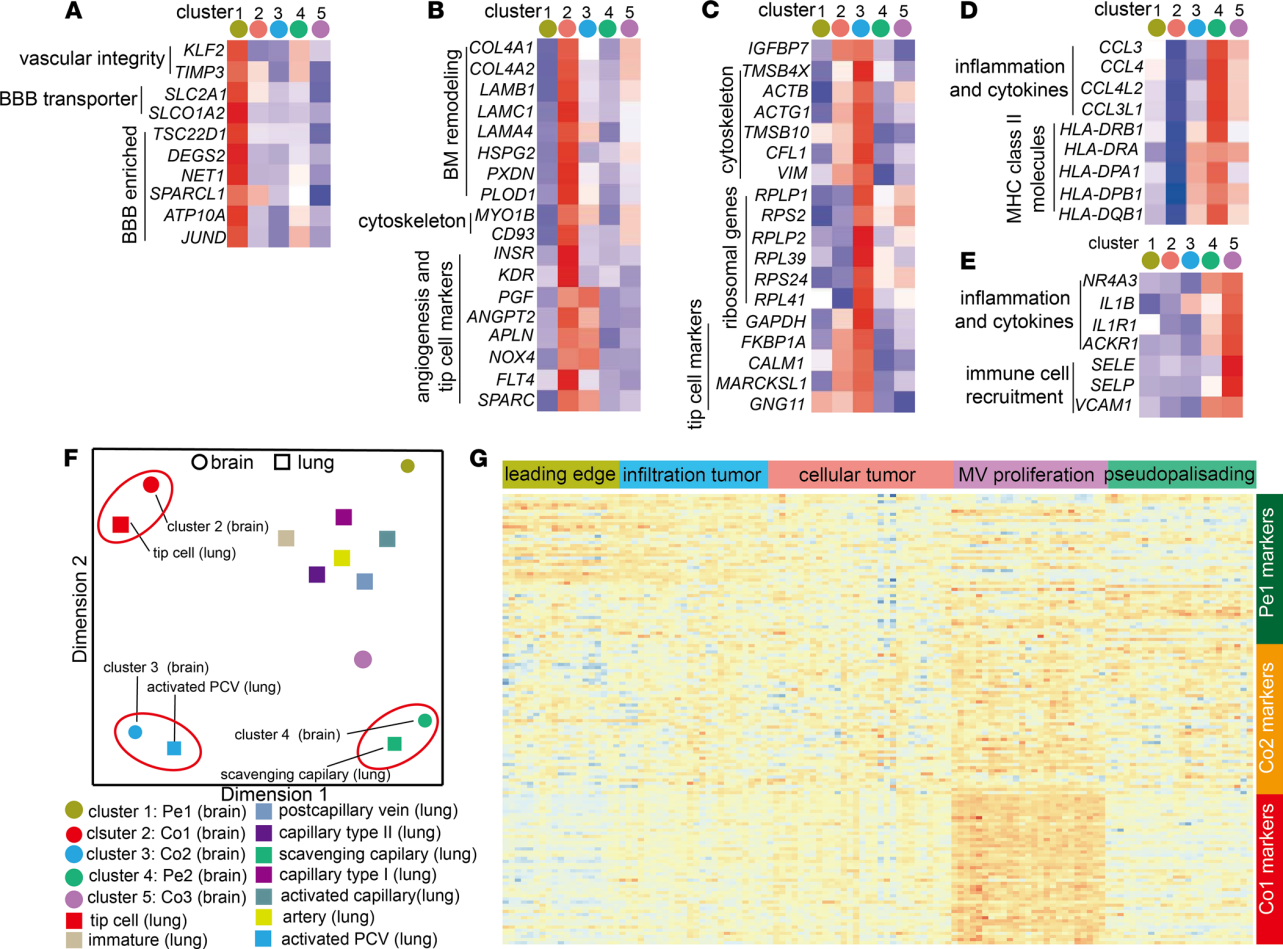
Characterization of different EC phenotypes. (**A**–**E**) Expression levels of selected marker genes of cluster 1 (Pe1) ECs (**A**), cluster 2 (Co1) ECs (**B**), cluster 3 (Co2) ECs (**C**), cluster 4 (Pe2) ECs (**D**), and cluster 5 (Co3) ECs (**E**). (**F**) Multidimensional scaling (MDS) on the Jaccard similarity coefficients of the top 50 marker gene sets among endothelial subclusters in brain (and GBM) and lung (and lung cancer). (**G**) The normalized expression of top 50 markers for Pe1 ECs, Co1 ECs, and Co2 ECs in Ivy GAP RNA-seq of distinct GBM-anatomic structures.

Cluster 1 ECs (referred to as peripheral EC type I [Pe1] because it originates mainly from the brain tissue surrounding the tumor mass) displayed a quiescent endothelial marker profile, as expected of normal brain ECs, and were characterized by a high expression level of genes implicated in vascular integrity (*KLF2*, *TIMP3*) and BBB function (*SLC2A1*, *SLCO1A2*) (Figure 3A, Supplemental Table 4, and Supplemental Figure 5). Several other genes, including *TSC22D1*, *DEGS2*, *ATP10A*, *SPARCL1*, and *NET1*, identified as top 50 markers for Pe1 ECs, were previously identified as BBB enriched genes in a recent study (5). Cluster 2 ECs (tumor core EC type I: Co1) resembled endothelial angiogenic phenotypes as they have been mapped in both developmental and tumor angiogenesis, and included for example a gene signature associated with vascular basement membrane remodeling (*COL4A1*, *COL4A2*, *LAMB1*, *LAMA4*, *HSPG2*, *PXDN*, *PLOD1*, *NID1*, *NID2*), cytoskeletal rearrangements (*CD93*, *MYO1B*, *SPARC*, *INSR*), angiogenic sprouting (*KDR*, *PGF*, *ANGPT2*, *NOX4*, *PTP4A3*, *FLT4*), and endothelial tip cell formation (*APLN*, *SOX4*, *ITGA5*, *PGF*, *NOTCH4*) (Figure 3B, Supplemental Table 4, and Supplemental Figure 5). Pe1 EC and Co1 EC phenotypes were validated by immunostaining CAVIN2 and HSPG2 on human specimen, respectively (Supplemental Figure 2, A–D).

Cluster 3 ECs (tumor core EC type II: Co2) showed upregulated genes involved in cytoskeletal and ribosomal protein expression, indicative of an intermediate phenotype (Figure 3C and Supplemental Table 5). Interestingly, Co2 ECs also expressed high level of genes identified as tip cell marker in previous study, including *FABP1A*, *CALM1*, *MARCHSL1* and *GNG11* (16). We also identified 2 EC clusters that exhibit immune-activated phenotypes: cluster 4 (peripheral EC type II: Pe2), expressing inflammatory cytokines (*CCL4*, *CCL3*) and genes involved in MHC-II-Mediated antigen presentation (*HLA-DRB1*, *HLA-DRA*, *HLA-DPA1*, *HLA-DPB1*, *HAL-DQB1*). This cluster was mainly derived from brain tissue surrounding the tumor core (Figure 3D, Supplemental Table 5). Cluster 5 (tumor core EC type III: Co3), were mainly derived from tumor core and were characterized by upregulation of immune-activated genes including *IL1B*, *ACKR1*, *SELE*, and *VACM1*, which are associated with inflammation and immune cell recruitment (Figure 3E and Supplemental Table 5). Both of these immune-activated EC types (Pe2 and Co3) predominantly consisted of cells originating from individual patients (Figure 2D), suggesting that they may represent individual differences in genetic background or systemic inflammation.

### Tumor ECs acquire similar phenotypes across different tumor types.

To explore whether tumor ECs have a similar phenotype across different tumor types, we analyzed our data set together with a recent publicly available scRNA-seq data set of ECs from human lung cancer and nonmalignant lung tissue using Jaccard similarity analysis (Figure 3F) (16). This analysis scored similarity of the top marker genes for all EC subclusters. As expected, Pe1 ECs from peripheral tissue exhibited a specialized phenotype and were clearly distinct from other EC subpopulations (Figure 3F). Interestingly, we found that Pe2 ECs in peripheral tissue express similar markers and gene sets, including antigen presentation and immune/inflammatory response, to so-called scavenging capillary ECs from lung (Figure 3E; Supplemental Figure 3, A and B; and Supplemental Table 6). Notably, in tumors, Co1 and Co2 ECs in GBM closely resemble the phenotypes of tip cells and the activated postcapillary vein (PCV) in lung cancer, respectively (Figure 3F and Supplemental Figure 3, C–F). Several congruent genes were identified as top 50 markers for both Co1 ECs in GBM and tip ECs in lung cancer (Supplemental Figure 3C and Supplemental Table 6). Gene sets involved in extracellular matrix organization, angiogenesis, and cell migration were commonly upregulated in both EC subtypes (Supplemental Figure 3D and Supplemental Table 6). Co2 ECs in GBM and activated PCV in lung cancer shared common markers and gene sets associated with mRNA translation and ribosome assembly (Supplemental Figure 3, E and F, and Supplemental Table 6). Taken together, these results suggest that, although GBM and lung cancer are distinct tumors, they comprise ECs exhibiting partially overlapping gene signatures.

### Distinct anatomical localization of individual EC cluster elucidated using reference atlases.

We next focused our attention on the phenotypes of Pe1 ECs, Co1 ECs, and Co2 ECs, which were represented in multiple patients. We analyzed the association of the EC clusters with anatomical location using the Ivy GAP database, which contains transcription profiles of human GBM anatomic regions including leading edge, infiltrating tumor region, cellular tumor region, microvascular proliferation, and pseudopalisading necrosis region (http://glioblastoma.alleninstitute.org/) (17). We found that normalized top 50 markers of Co1 ECs were enriched in microvascular proliferation regions and downregulated in leading edge region (Figure 3G). In contrast, Pe1 EC markers, including *SLCO1A2*, *ANXA3*, and *TSC22D1*, were enriched in leading edge and infiltrating tumor regions (Figure 3G). These results confirm the distinct anatomical localization of ECs with different phenotypes using an independent data set.

### ECs in GBM are distinct from ECs in peripheral brain tissue.

**Figure 4:**
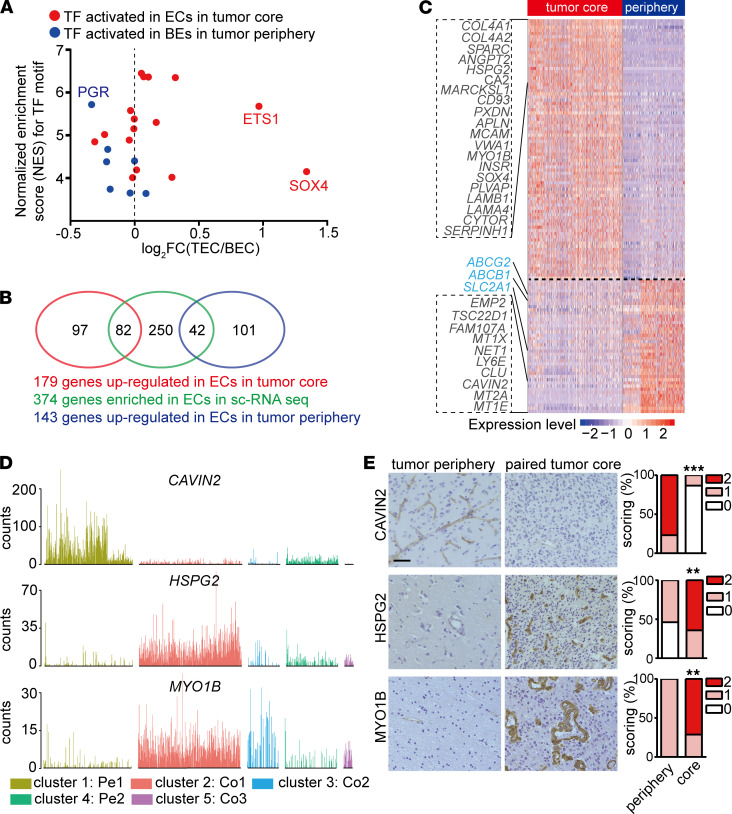
ECs in tumor core are distinct from ECs in peripheral brain tissue. (**A**) Plot of transcription factor activity score estimated by SCENIC to fold change of their expression between ECs in periphery and tumor core. Red/blue dot corresponds to transcription factor activated in ECs in the tumor core or tumor periphery, respectively. (**B**) Venn diagram illustrating the overlaps of 374 EC-enriched genes with differentially expressed genes between ECs in tumor core and tumor periphery. (**C**) Heatmap showing differentially expressed EC-enriched genes between ECs in the tumor core and peripheral brain tissue. (**D**) Bar plots of *CAVIN2*, *HSPG2*, and *MYO1B* among different EC subclusters. (**E**) IHC staining and quantification of CAVIN2, HSPG2, and MYO1B in human GBM tumor core and paired peripheral brain tissue (CAVIN2, *n =* 13; HSPG2, *n =* 13; MYO1B, *n =* 14). Staining was scored semiquantitatively on scale from 0 to 2 based on proportional of vessels stained (Wilcoxon test, **P < 0.01, ***P < 0.001). Scale bar: 50 μm.

In order to explore which transcription factors may regulate the distinct phenotype of ECs in tumor periphery and tumor core, we employed single-cell regulatory network inference and clustering (SCENIC; ref. 18) to evaluate the activated transcription factors in Pe1 and tumor core ECs including both Co1 and Co2 (Supplemental Table 7). This identified several activated transcription factors in ECs in tumor periphery and tumor core (Figure 4A and Supplemental Table 7). Notably, *SOX4* and *ETS1*, which were identified as activated transcription factors in ECs in the tumor core, were also upregulated in ECs in the tumor core (Figure 4C). Interestingly, elevated *ETS1* expression was also observed in vasculature of the normal isocitrate dehydrogenase (IDH-WT) lower-grade gliomas (LGGs) compared with vasculature of IDH-mutated LGGs in our previous study (9). SOX4 has been suggested to promote tumor angiogenesis through CXCR4 and endothelin-1 in breast and hepatocellular tumors, respectively, in 2 recent studies (19, 20).

To evaluate global metabolic alteration in ECs, we performed GSVA with metabolic gene sets (21) for ECs in the tumor peripheral tissue and tumor core. This analysis revealed that ECs in the tumor core displayed an upregulation of glycolysis, citric acid cycle, and oxidative phosphorylation gene expression signatures (Supplemental Figure 4A), similar to what was previously observed in ECs from human lung cancer as compared with nonmalignant lung (22). Considering that ECs are highly glycolysis-addicted cells, high glycolysis in tumor ECs may reflect high demand of energy requirements for angiogenesis in the tumor microenvironment. Indeed, therapeutically inhibiting glycolysis could inhibit tumor angiogenesis and normalize tumor vessels (23). Upregulation of oxidative phosphorylation and the tricarboxylic acid (TCA) cycle are noteworthy, and this observation is in line with a recent study showing that the oxidative phosphorylation is necessary for angiogenesis (24). Inhibition of oxidative phosphorylation by ablation of a subunit in respiratory chain complex III in ECs leads to diminished EC proliferation and impairment in retinal and tumor angiogenesis, accompanied by decreased amino acid levels, without affecting anabolism or nucleotide levels (24). To further explore which metabolic genes and gene sets were regulated in ECs in tumors, we performed differential gene expression analysis and constructed a map of pathways involved in central carbon metabolism (Supplemental Figure 4B). The results confirmed upregulation of genes in metabolic pathways supporting biomass synthesis, including glycolysis, citrate cycle, oxidative phosphorylation, and nucleotide synthesis and downregulation of genes in glutamate metabolism in ECs in the tumor core (Supplemental Figure 4B). Taken together, our results indicate that ECs in tumors are associated with an altered metabolic transcriptome signature characterized by increased expression of genes involved in glycolysis, citrate cycle, and oxidative phosphorylation.

### Identification of GBM endothelial markers and angiogenic regulators.

In order to identify angiogenic regulators and to discover markers discriminating GBM and surrounding vasculature, we first identified 374 EC-enriched genes by comparing ECs cells with other CD31^+^ cell types in our data sets, and we subsequently compared the expression of the 374 EC-enriched genes between ECs in tumor core and tumor periphery (Figure 4, B and C, and Supplemental Table 8). Forty-two EC-enriched genes, including BBB-related transporters, were downregulated in ECs from the tumor core (Figure 4, B and C). Downregulation of CAVIN2 in tumor core vasculature was validated by immunostaining in human specimens (Figure 4, D and E). Eighty-two EC-enriched genes were upregulated in ECs in the tumor core, including collagens (and their modifying enzymes; *COL4A1*, *COL4A2*, *PXDN*), laminins (*LAMB1*, *LAMA4*, *LAMC1*), matricellular proteins (*SPARC*, *HSPG2*), adhesion molecules (*CD93*, *MCAM*, *ITGA5*, *ITGA1*, *ITGB1*), a vascular permeability marker (*PLVAP*), and angiogenic molecules (*ANGPT2*, *HSPG2*, *APLN*, *KDR*). Upregulation of HSPG2 and MYO1B in vasculature in the tumor core were validated by immunostaining in human specimens (Figure 4, D and E).

### ECs in GBM are associated with a partially intact BBB phenotype characterized by downregulation of transporter genes and upregulation of transcytosis gene.

**Figure 5:**
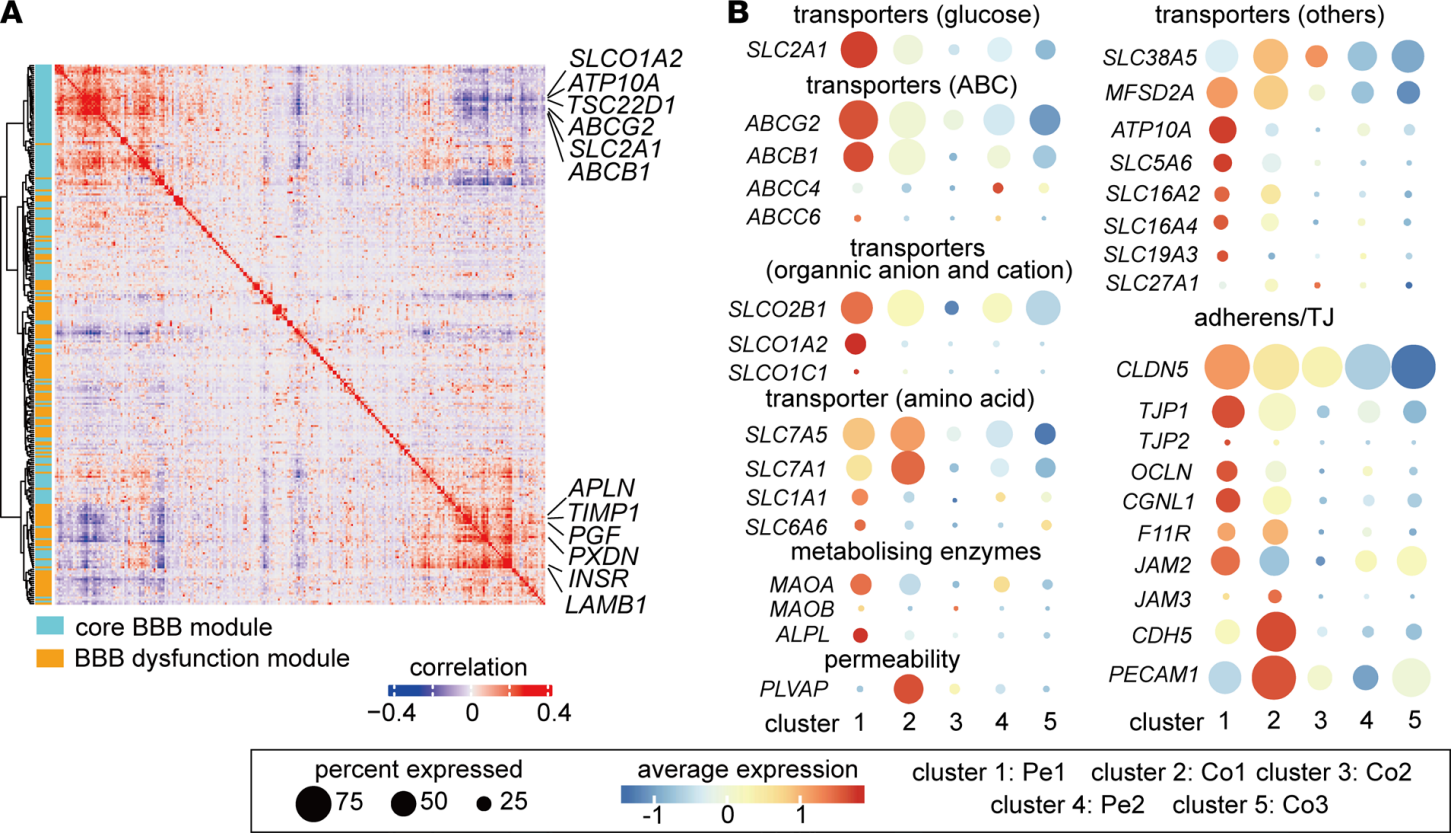
ECs in GBM are associated with compromised BBB phenotype. (**A**) Correlation plot showing correlation coefficient of genes in BBB and BBB dysfunctional module. (**B**) Dot plot showing differential expression patterns of different transporters, metabolizing enzymes, adherens/tight junctions, and permeability genes implicated in BBB functions among different clusters.

Emerging studies suggest that the BBB is disrupted during tumor progression (reviewed in ref. 6). However, whether and to what extent BBB alterations in GBM ECs can be documented at the single-cell level remains unknown. We therefore compared our data with a recently reported BBB dysfunction module, which compiles a set of genes that are upregulated in ECs in at least 3 of 4 mouse brain disease models where the BBB has been disrupted, including stroke, multiple sclerosis, traumatic brain injury, and seizure (5, 25). The BBB core module comprises a group of genes enriched in brain ECs compared with ECs from peripheral organs (5). Coexpression analysis of the BBB dysfunction module with the BBB core module revealed that expression of key transporters in the BBB, including *SLC2A1*, *ABCG2*, *ATP10A*, *SLCO1A2*, and *ABCB1*, anticorrelated with the expression of genes involved in tip cell formation and extracellular matrix remodeling, including *APLN*, *LAMB1*, *PCDN*, and *TIMP1* (Figure 5A and Supplemental Figure 5). APLN was identified as a marker for sprouting ECs, and it is required for tip cell formation and sprouting (26, 27). These observations and this coexpression analysis suggest that tumor ECs with tip cell phenotypes might have decreased BBB property during tumor angiogenesis.

**Figure 6:**
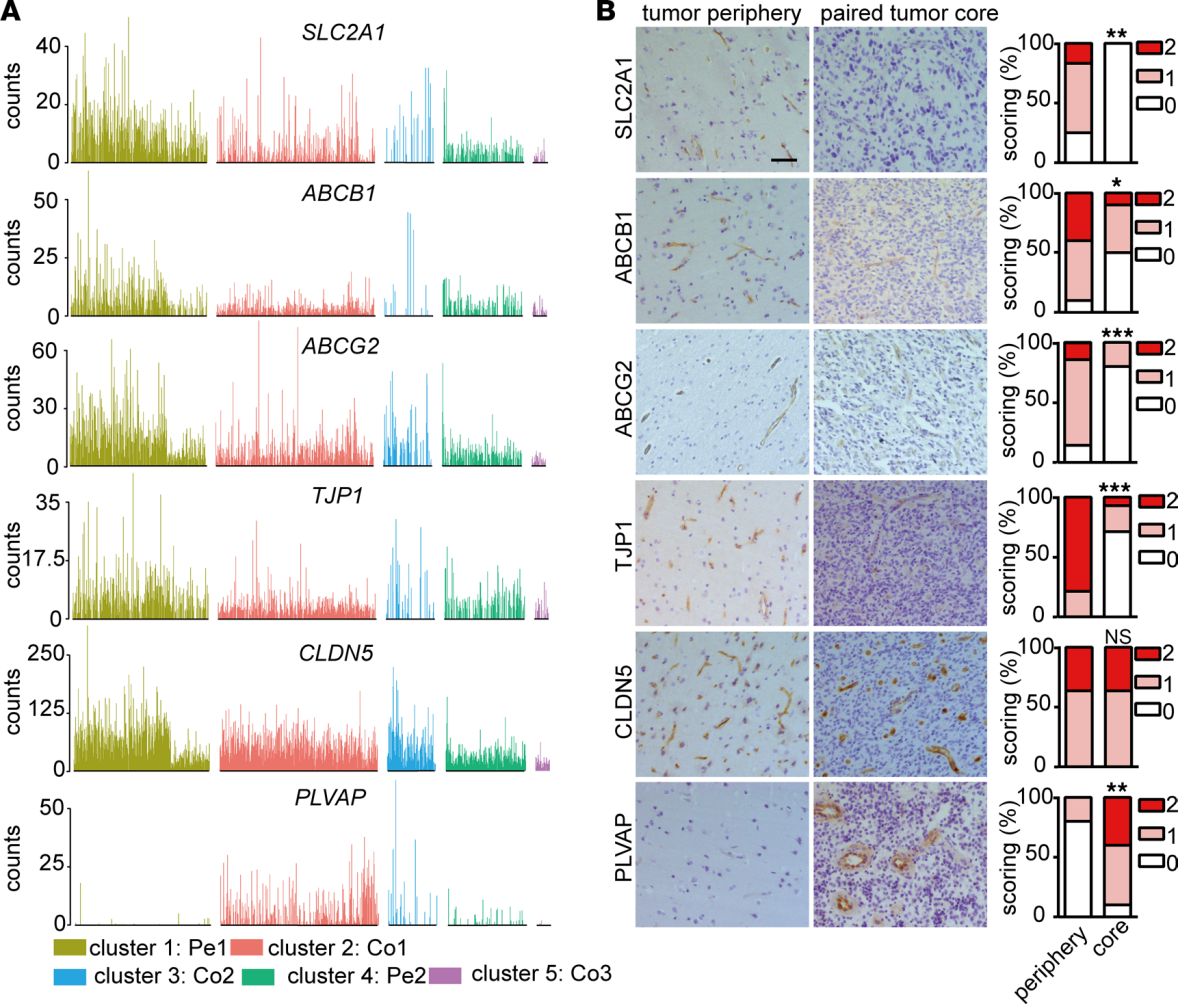
The expression of BBB-related genes in EC subclusters. (**A**) Bar plots of *SLC2A1*, *ABCB1*, *ABCG2*, *TJP1*, *CLDN5*, and *PLVAP* in different EC subclusters. (**B**) IHC staining and quantification of SLC2A1, ABCB1, ABCG2, TJP1, CLDN5, and PLVAP in peripheral brain tissue and paired GBM (SLC2A1, *n =* 12; ABCB1, *n =* 10; ABCG2, *n =* 14; TJP, *n =* 14; CLDN5, *n =* 11; PLVAP, *n =* 10). The stainings were scored semiquantitatively on scale from 0 to 2 based on proportional of vessels stained (Wilcoxon test, *P < 0.05, **P < 0.01, ***P < 0.001). Scale bar: 50 μm.

Specialization of the endothelium, which is characterized by formation of tight junctions with neighboring ECs together with expression of BBB-related transporters, is a key feature of the BBB (6). Notably, we observed a heterogeneous expression of junctions and transporters in different EC clusters (Figure 5B). While expression of the tight-junction gene *CLDN5* was similar in Pe1 ECs and Co1 ECs (Figure 5B and Figure 6, A and B), some adherens junction mRNAs, including VE-cadherin (encoded by *CDH5*) and *CD31*, were upregulated in ECs in the tumor core compared with peripheral ECs (Figure 5B). Interestingly, several BBB-related transporters, including *SLC2A1*, *ABCG2*, *ABCB1*, *SLCO1A2*, and *ATP10A* were significantly decreased in ECs in tumor core (Figure 5B and Figure 6, A and B). Notably, however, BBB-related transporters were only partially lost in ECs in the tumor core, suggesting that BBB is partially intact. In accordance with increased permeability of GBM vasculature, we observed a higher level of expression of *PLVAP* in ECs in the tumor core compared with ECs in peripheral tissue. PLVAP is a marker of vascular fenestration and associated with vascular leakage (Figure 5B and Figure 6, A and B) (28). Our data indicate that ECs in GBM are associated with a partially intact BBB phenotype characterized by downregulation of transporter genes and upregulation of a gene implicated in fenestration and transcytosis.

## Discussion

Previous bulk analysis–based transcriptomic studies have revealed limited insights into the characteristics of ECs in normal brain and GBM (7, 8). In bulk RNA analysis, cellular heterogeneity gets lost in the average of gene expression from all cells, and contaminations from other cell types cannot be deduced, together posing severe limitations to interpretations and the possibilities for correctly assigning EC-specific changes and their heterogeneity within the EC population. Indeed, in our previous work (8), we identified a distinct gene signature associated with GBM vessels, which was composed of genes expressed in all cell types enriched in the vasculature, including ECs and mural cells. Here, by performing scRNA-seq, we characterized brain and GBM ECs in more detail and identified 5 distinct EC phenotypes in GBM and peripheral brain tissue with normal histology. ECs in tumor peripheral tissue have a quiescence phenotype (cluster 1, Pe1), characterized by high expression of BBB enriched genes including *SLC2A1* and *KLF2*. KLF2, a key transcription factor orchestrating a network of genes that promotes EC quiescence in response to flow (29), is one of the top 10 enriched genes in brain EC cluster. GLUT1, encoded by *SLC2A1*, is highly expressed in BBB ECs and facilitates glucose transport over BBB (25). Depletion of GLUT1 in adult brain ECs leads to activation of inflammatory and extracellular matrix–related gene sets (25).

We also identified ECs with angiogenic phenotype (cluster 2, Co1) that expressed a high level of genes involved in basement membrane remodeling, cytoskeleton rearrangement, angiogenesis, and tip cell formation. Notably, CD93 is one of the top 10 enriched genes in Co1 ECs and had been identified as key regulator orchestrating cytoskeleton and matrix organization for ECs during angiogenesis in our previous studies (30). Genes encoding collagens (*COL4A1*, *COL4A2*), collagen-modifying enzyme (*PXDN*), and other components of the basement membrane (*LAMB1*, *HSPG2*) also ranked in the top 10 most enriched Co1 EC markers, indicating that extensive matrix remodeling occurs in GBM during tumor angiogenesis. By integrating recently published scRNA-seq data of ECs from lung cancer and control tissue into our analysis (16), we found that angiogenic Co1 ECs in GBM and tip ECs in lung cancer share markers and enriched gene sets. This observation suggests that, although the tumor types and the original vessels are distinct, pathological tumor angiogenesis may be modulated through similar mechanisms.

It is a common belief that the BBB is disrupted during tumor progression in the brain. Our scRNA-seq data indeed show that tumor ECs in GBM have a partially intact BBB phenotype, characterized by downregulation of transporter genes, whereas the expression of junctional molecules remained normal or was increased. ECs in GBM express a high level of *PLVAP*, which is a vascular marker of BBB disruption. PLVAP expression is absent in the brain vasculature, with the exception of the choroid plexus and circumventricular organs where the endothelium is fenestrated to allow filtration of plasma for CSF production and passage of hormones (31). However, PLVAP is induced in pathological conditions in the brain and associated with vascular leakage (28). PLVAP is a key regulator of vascular permeability and promotes transcytosis in ECs by forming the diaphragms of caveolae, fenestrae, and transendothelial channels (28). Vascular leakage is a hallmark of GBMs and may be induced by 2 main pathways: (a) increased paracellular transport by altering the tight junctions between ECs and/or (b) increased transcellular transport by altering vesicular transcytosis. In contrast to upregulation of *PLVAP* in Co1 ECs, the expression of tight-junction molecules, including *CLDN5*, was similar between ECs in the periphery and tumor core, highlighting an important role of transcellular pathways for BBB breakdown and the edema formation observed in GBM.

Several key BBB transporters, including *SLC2A1*, *ABCG2*, *ABCB1*, *SLCO1A2*, and *ATP10A*, were highly expressed in the ECs of the brain tissue surrounding the GBM core. P-glycoprotein (P-gp, encoded by *ABCB1*) and breast cancer resistance protein (BCRP, encoded by *ABCG2*) are ATP-binding cassette transporters that, together, mediated efflux of xenobiotics, including temozolomide and other low–molecular weight anticancer drugs from the endothelium away from the neuroparenchymal space (32).

Our findings showing both preservation and heterogeneity of BBB phenotypes in GBM ECs have important clinical implications for GBM treatment, since GBMs comprise heterogeneous glioma stem cells (GSCs), including proneural GSCs (pGSCs) and mesenchymal GSCs (mGSCs), with different transcriptomic subtypes and distinct anatomic localization (17, 33). pGSCs are enriched in the tumor leading edge and infiltrating regions (17). Thus, it is of importance to inhibit BBB transporters (*ABCB1* and *ABCG2*) simultaneously when targeting pGSCs. For mGSC targeting, drugs with high transcytosis over ECs are likely to be more enriched in the tumor core, where mGSCs were enriched (17).

In conclusion, we have developed a single-cell transcriptome resource to aid understanding of the characteristic of CNS ECs and their alteration in GBM. This resource may provide vital information of relevance for drug delivery and intratumoral distribution in GBM, and it may facilitate the design of rational therapeutic regimens.

## Methods

### Isolation of CD31^+^ ECs from human GBM and peritumoral tissue.

We collected surgical tissues from 4 patients with GBM to isolate ECs for scRNA-seq. The GBM tumors were located in the right parietal lobe for patient 1, the left temporal lobe for patient 2, the right occipital lobe for patient 3, and the right frontal lobe for patient 4 (Supplemental Table 1). From each patient, we collected 2 separate tissue samples: one originating from the tumor core and another from the peritumoral space. For each sample, the tumor core and peritumor tissue were processed separately. Tissue samples were immediately transported to the research facility in order to start sample dissociation within 2 hours of resection. Tissue samples were mechanically dissociated and then processed into single-cell suspension using tumor dissociation kit (130-095-929, Miltenyi Biotec) for tumor core tissue, using an adult brain dissociation kit (130-107-677, Miltenyi Biotec) for peritumor tissue. Single-cell suspensions were run through debris removal solutions to remove the myelin debris, and they then proceeded to remove RBCs according to manufacturer’s specifications. CD31^+^ selection for EC enrichment was performed using Dynabeads (11155D, Invitrogen). For further processing for scRNA-seq, the samples were resuspended in DPBS containing 0.04% BSA. The number of cells and fractions of live cells in suspension was counted, and the volume of suspension containing the required number of live cells was used for scRNA-seq as described below.

### Single-cell, droplet-based scRNA-seq, quality control (QC) and data processing.

scRNA-seq libraries were prepared using Chromium Single Cell Reagent Kit (10X Genomics). The libraries were then pooled and sequenced on NovaSeq 6000 (Illumina), using NovaSeq Control Software v1.6.0. The raw sequence data were processed using Cell Ranger software (v.3.0.1). The reads were aligned to human genome GRCh38, and a gene count matrix was generated for each sample. The raw count data were then loaded into Seurat package (v3.1.1) for QC, filtering, normalization, Uniform Manifold Approximation and Projection (UMAP) visualization, and clustering (15). The cells that have mitochondrial genes greater than 10% or have fewer than 200 detected genes were filtered out. A scale factor of 10,000 was used to normalize all the remaining cells. To correct for the batch effect between different samples, and the reciprocal principal component analysis (RPCA) method in the Seurat package was applied to integrate the complete data set. The genes enriched in each cluster were identified using FindAllMarkers function in Seurat. It applies a Wilcoxon Rank Sum test and then performs multiple test correction using the Bonferroni method. The multiple-test corrected *P* < 0.05 was used as cut-off for significance.

### Similarity analysis for different tumor ECs.

To illustrate the similarity of the different tumor EC groups from our study with previous published lung tumor EC subtypes (16), we applied Jaccard similarity analysis. The top 50 enriched genes from each cluster were compared, and a pair-wise Jaccard similarity coefficients matrix was calculated. The result matrix was then visualized in 2D using the classical multidimensional scaling method in R (version 3.6.1)

### Analysis of anatomical localization of individual EC cluster marker genes using Ivy GAP database.

The transcriptome data from different human GBM anatomic regions, including leading edge, infiltrating tumor region, cellular tumor region, microvascular proliferation, and pseudopalisading necrosis region, were obtained from the Ivy GAP database (http://glioblastoma.alleninstitute.org). Due to the heterogeneity of vascular abundance in different anatomical locations, a direct comparison of different EC cluster markers is inappropriate. Therefore, the original marker gene expression values were normalized by a microvascular score, which was calculated by vascular enriched genes to estimate the relative abundance of the vasculature in each sample, as described previously (9). The normalized expression of the marker genes for different EC clusters in distinct anatomical locations were shown by heatmap as Figure 3G.

### Gene set variation analysis (GSVA).

To identify the gene sets with significant changes in the tumor clusters, we applied GSVA using GSVA package (version 1.32.0). The metabolic gene sets obtained from a published study (21), were tested using the R limma package (version 3.40.6). The individual *P* values from testing on multiple gene sets were adjusted using the Benjamini-Hochberg method and the gene sets with corrected *P* < 0.05 were identified as significant.

### SCENIC analysis.

To identify the transcription factors that regulate the tumor EC clusters, SCENIC analysis was performed using the RcisTarget package (version 1.4.0) using default settings. It identifies overrepresented transcription factor binding motifs on a gene list, and those motifs were then annotated to transcription factors.

### Core BBB and BBB dysfunction module.

A core BBB module gene set was generated according to the previous study; it compared the transcriptome of mouse brain ECs with ECs in peripheral organs, including kidney, lung, heart, and liver (5). The core BBB module gene set comprises 162 genes that were selected based on following criteria: (a) at least 100 counts per million (CPM) mapped reads detected in brain ECs; (b) more than 2-fold upregulation (and *P* < 0.05) in brain ECs compared with ECs from other 4 organs individually; and (c) genes with expression in brain ECs no less than brain vasculature (exclude contamination from mural cells). In total, 155 of the 162 genes have human homologs, and 147 of them were detected in our data set.

The BBB dysfunction module gene set comprises 136 genes that are upregulated in ECs in at least 3 of 4 mouse diseased models, including stroke, multiple sclerosis, traumatic brain injury, and seizure, in a previous study (5). In total, 131 genes have human homologs, and 128 of them were found in our data set.

### Identification markers for control and GBM vasculature.

Direct comparison of ECs with other CD31^+^ cell types yield to 374 EC-enriched genes (Bonferroni-corrected *P* < 0.05). The expression of 374 EC-enriched genes were compared between ECs in the periphery and tumor core, listed in Supplemental Table 8.

### HE staining analysis.

Patient samples were fixed in 4% formalin and embedded in paraffin, followed by section and staining with H&E. Then the sections were examined for the presence of tumors.

### IHC staining and quantification.

IHC was performed on 6 μm sections of formalin-fixed, paraffin-embedded tissues. The sections were deparaffinized and dehydrated, and antigen retrieval followed. Then, the sections incubated with primary antibody toward CD31 (AF806, R&D Systems), CAVIN2 (NBP1-44090, Novus), HSPG2 (AF2364, R&D Systems), MYO1B (ab194356, Abcam), ABCG2 (ab24115, Abcam), SLC2A1 (HPA058494, Sigma-Aldrich), ABCB1 (HPA002199, Sigma-Aldrich), TJP1 (HPA001637, Sigma-Aldrich), CLDN5 (341600, Invitrogen), and PLVAP (NBP1-83911, Novus). All quantification was done by 2 individual scientists in a blinded fashion.

### Immunofluorescence staining of patient samples.

Immunofluorescence was performed on 6 μm sections of snap-frozen tissue embedded in OCT (Tissue-Tek Sakura). The sections were incubated with primary antibody toward CD31 (AF806, R&D Systems), CAVIN2 (NBP1-44090, Novus), and HSPG2 (NBP2-44448, Novus) overnight at 4°C, followed by incubation with the secondary antibody and nuclear staining with Hoechst 33258 (Sigma-Aldrich). The slides were then mounted with Fluoromount (Sigma-Aldrich). All the images were acquired by Axio Imager upright microscope (Zeiss).

### Data availability.

The scRNA-seq raw sequencing data and also processed counts data are available in the NCBI Gene Expression Omnibus under accession no. GSE162631.

### Statistics.

The IHC stainings were quantified and analyzed using the 2-tailed Wilcoxon test. A *P* value of less than 0.05 was considered significant.

### Study approval.

Samples were obtained following informed consent, under the auspices of the Tangdu Hospital of Fourth Military Medical University (THFMMU, 2019-0166). Ethical permit of the use of patient samples was granted by the ethics committee of Shaanxi Normal University, and informed consent was obtained from all subjects (Supplemental Table 1). Any information that might disclose the identity of the subjects has been omitted.

## Author contributions

LZ, AD, LW and CB designed research, analyzed and interpreted data, and wrote the manuscript. YX, L. He, RL, and Yanyu Zhang performed research and collected, analyzed, and interpreted data. YX collected data, performed IHC, data analysis and manuscript preparation. L. He performed bioinformatics, data analysis and manuscript preparation. RL performed sample annotation, data analysis, data interpretation and manuscript preparation. HC, QH, MC, BL, QC, JW, YJ, YH, and L. Han performed research. Yong Zhang, HH, and LU participated in data analysis. Author order reflects the relative size and importance of the contributions to the project and manuscript.

## Supplementary Material

Supplemental data

Supplemental Table 1

Supplemental Table 2

Supplemental Table 3

Supplemental Table 4

Supplemental Table 5

Supplemental Table 6

Supplemental Table 7

Supplemental Table 8

## Version 1 07/06/2021

In-Press Preview

## Version 2 08/09/2021

Electronic publication
