# Supplementary material for: Key molecular alterations in endothelial cells in human glioblastoma uncovered through single-cell RNA sequencing
Source: JCI Insight. 2021 Aug 9;6(15):e150861. doi: 10.1172/jci.insight.150861 (PMC8410070; doi:10.1172/jci.insight.150861)
Supplement: Supplemental data [file jciinsight-6-150861-s058.pdf]

Supplementary Information

Key Molecular Alterations in Endothelial Cells in Human Glioblastoma Uncovered through Single-Cell RNA Sequencing

Supplementary Figures

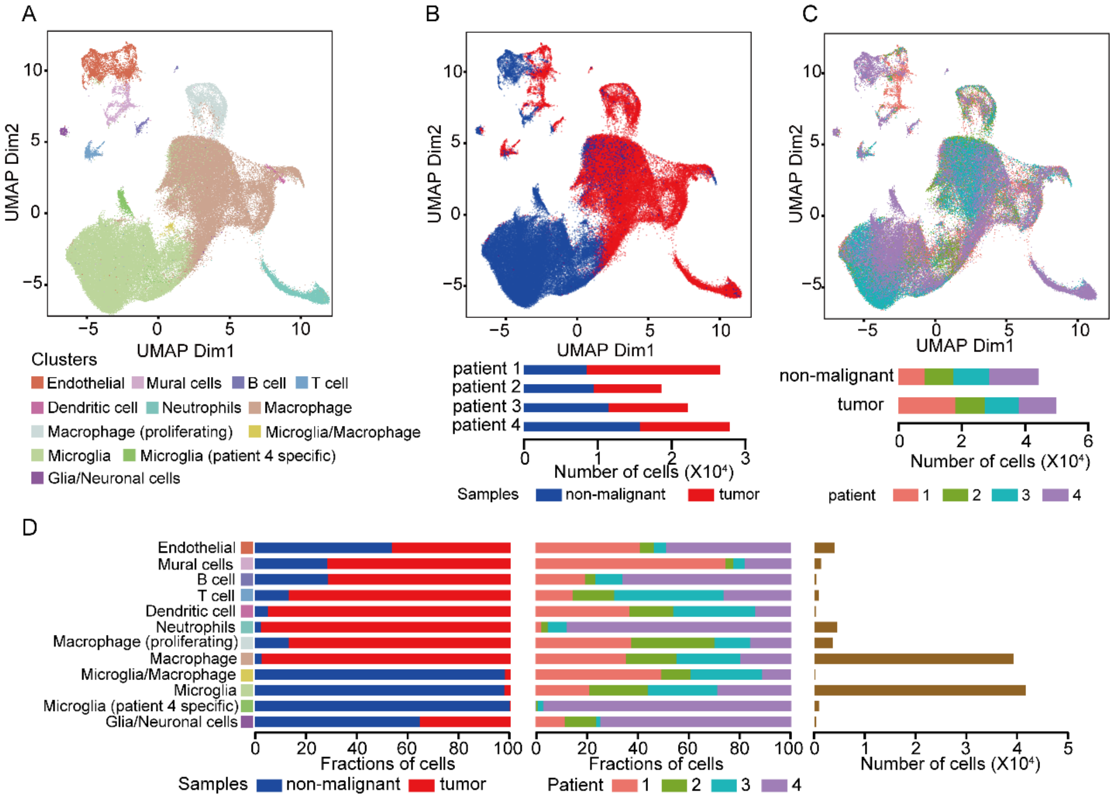

Figure S1 Overview of the CD31-MACS enriched single cells from GBM and peripheral brain samples. (A-C) UMAP of all cells, colored by clusters (A) or sample type of origin (B) or individual patient (C). (D) Relative contribution of all cells from sample origin type (left) or individual patient (middle) or the number of cells in each cluster (right).

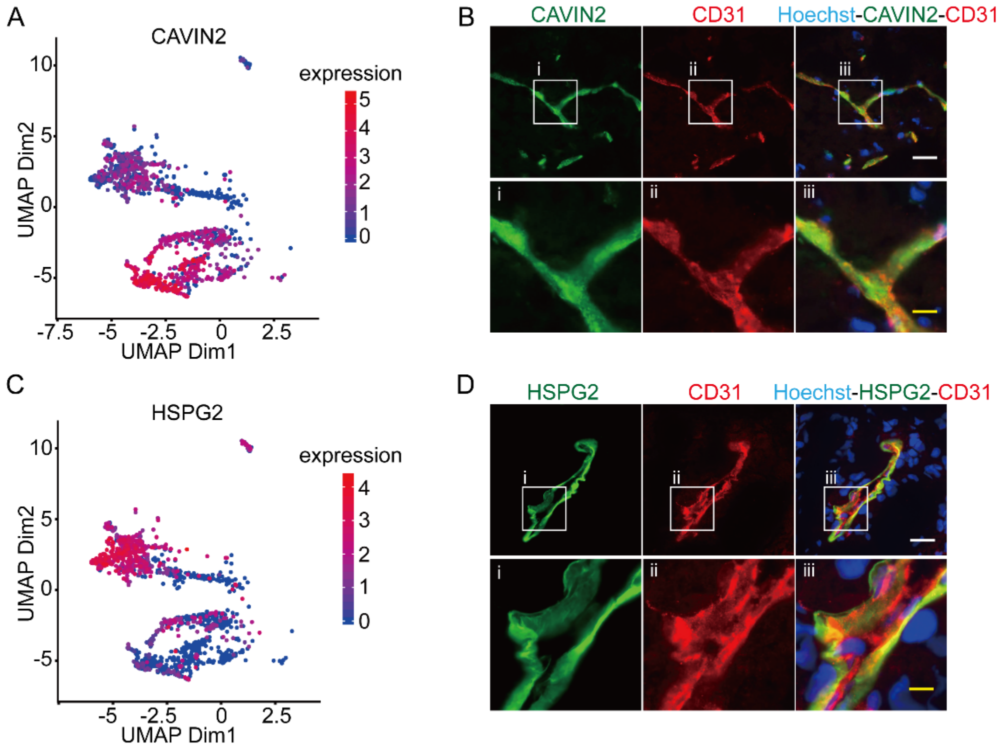

Figure S2 Validation of cluster 1 (Pe1) EC phenotype and cluster 2 (Co1) phenotype. (A) UMAP plot of all cells indicating the level of expression of *CAVIN2*. (B) Immuno-staining CAVIN2 and CD31 on human brain tissue. (C) UMAP plot of all cells indicating the level of expression of *HSPG2*. (D) Immuno-staining HSPG2 and CD31 on human GBM tissue. (White scale bar=20µm, yellow scale bar=6um)

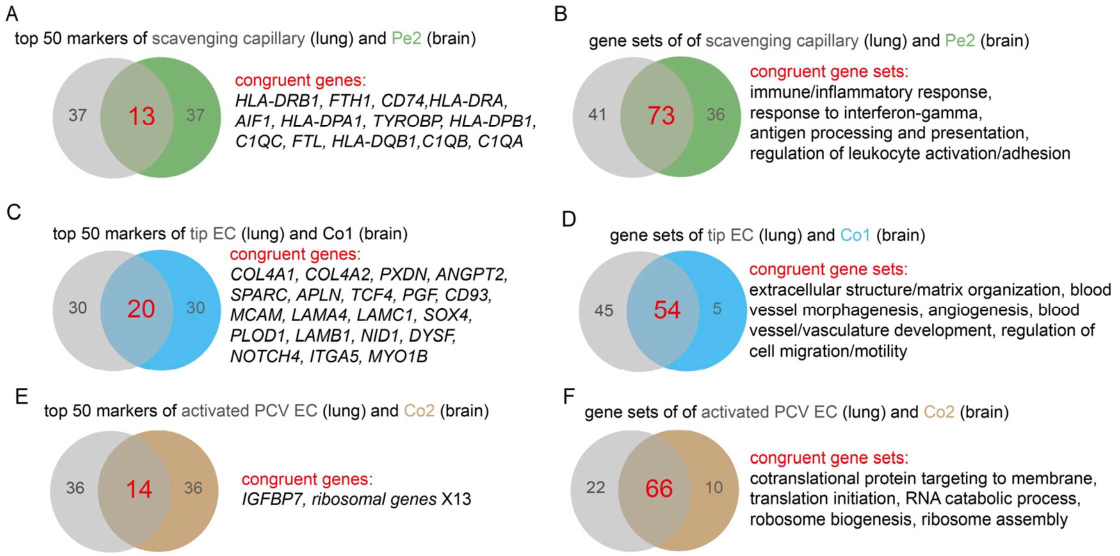

Figure S3 Phenotypes of tumor ECs across different tumor types: (A-F) List of congruent genes of top 50 markers or congruent gene-sets derived from top markers of scavenging capillary (lung) and cluster 4 (Pe2) ECs (brain) (A, B), or tip ECs (lung) and cluster 2 (Co1) ECs (brain) (E, F), or activated PCV ECs (lung) and cluster 3 (Co2) ECs (brain) (G, H).

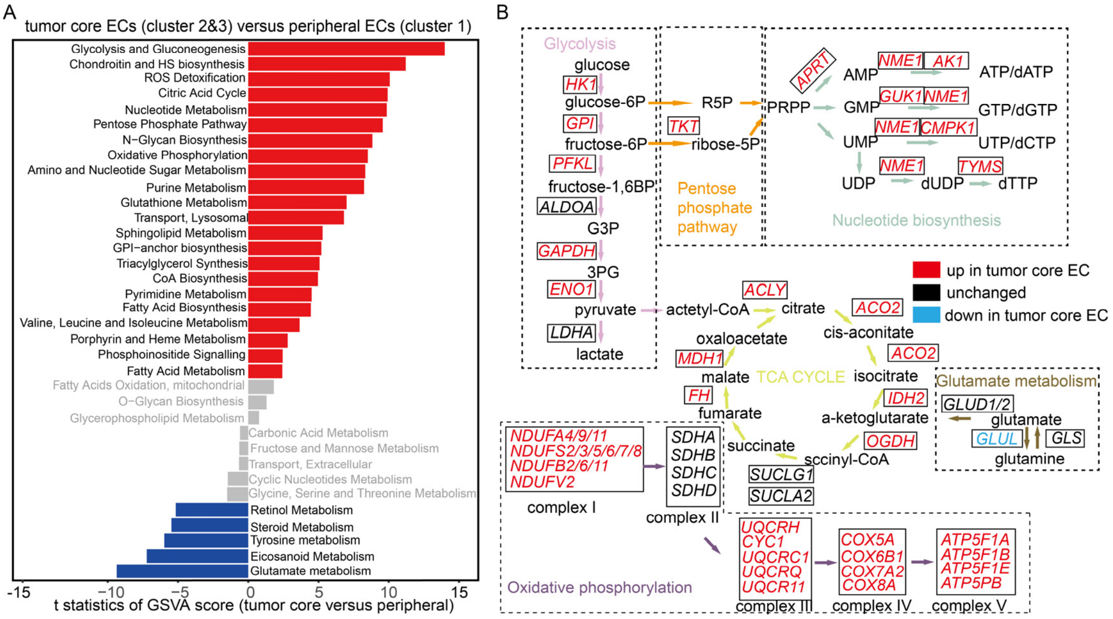

Figure S4 ECs in tumor core are associated with an altered metabolic transcriptome signature: (A) Difference of metabolic gene set between ECs in tumor core or tumor peripheral tissue scored by GSVA. (B) Map of altered central carbon metabolic pathways in ECs. Red color indicated up-regulation in ECs in tumor, blue color indicated down-regulated in ECs in tumor, and gray unchanged expression.

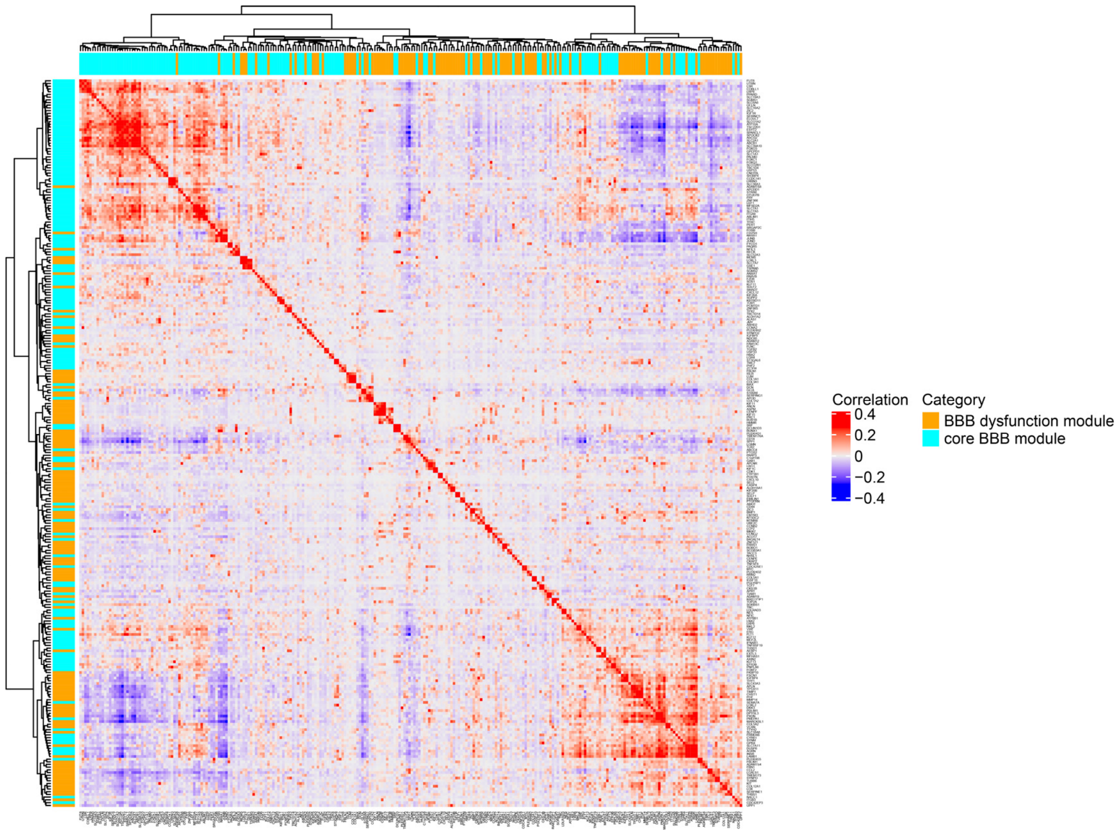

Figure S5 Correlation plot showing correlation co-efficient of genes in BBB and BBB dysfunctional module.
