# Supplementary material for: Key molecular alterations in endothelial cells in human glioblastoma uncovered through single-cell RNA sequencing
Source: JCI Insight. 2021 Aug 9;6(15):e150861. doi: 10.1172/jci.insight.150861 (PMC8410070; doi:10.1172/jci.insight.150861)
Supplement: Supplemental Table 5 [file jciinsight-6-150861-s063.pdf]

Table S5 Molecular phenotypes of freshly isolated ECs from GBM and tumor peripheral tissue

Assigned phenotype	Nr.	Expressed genes typical of
peripheral endothelial cell type I (Pe1)	cluster 1	• Vascular integrity: KLF2[1]; TIMP3[2]<br>• BBB Transporter: SLC2A1[3]; SLCO1A2[4]<br>• BBB enriched genes: TSC22D1; DEGS2; NET1; SPARCL1; ATP10A; JUND[5]
tumor core endothelial cell type I (Co1)	cluster 2	• Basement membrane remodeling: COL4A1[6]; COL4A2; LAMB1[7]; LAMC1[8]; LAMA4[9]; HSPG2[10];PXDN, PLOD1; NID1; NID2[11]<br>• Cell migration and cytoskeleton associated genes: MYO1B[12]; CD93[13, 14], INSR[15]; SPARC[16], RASGRP3[17];<br>• Angiogenesis: KDR, PGF, ANGPT2, APLN[18], NOX4[19]; PTP4A3[20]; FLT4[21]<br>• Vascular remodeling: NOTCH4[22]; UNC5B[23]<br>• Tip cell markers: COL4A1; PXDN; COL4A2; NID1; CD93; ANGPT2; LAMB1; MCAM; TCF4; LAMC1; LAMA4; MYO1B; SPARC; PGF; NOTCH4; SOX4; PLOD1; ITGA5; APLN[24]
tumor core endothelial cell type II (Co2)	cluster 3	• Cytoskeleton organization: TMSB4X; ACTB; ACTG1; TMSB10; VIM [25]; PFN1[26]; MYL6; MARCKSL1; ARPC2; MYL12B; CFL1[27]<br>• Ribosomal genes: RPLP1; RPS2; RPLP2; RPL39; RPS24; RPL41; RPL6; RPL13; RPL8; RPL35; RPLS8; RPS28; RPS12; RPS26; RPS6; RPL36; RPL10; RPL15; RPS4X; RPLP0<br>• Tip cell markers: GAPDH; ACTG1; CFL1; VIM; FKBP1A; CALM1; MARCKSL1; GNG11[24]
peripheral endothelial cell type I (Pe1)	cluster 4	• Inflammation and cytokine: CCL4; CCL4L2; CCL3L1; ACKR1[28]; CCL3[29]<br>• MHC class II molecules: HLA-DRB1: HLA-DRA; HLA-DPA1; HLA-DPB1; HLA-DQB1
tumor core endothelial cell type III (Co3)	cluster 5	• Inflammation and cytokines: NR4A3; IL1B; IL1R1; ACKR1[28, 30, 31]<br>• Immune cell recruitment: SELE; SELP;VCAM1[32]

1. Sangwung, P., et al., *KLF2 and KLF4 control endothelial identity and vascular integrity.* JCI Insight, 2017. **2**(4): p. e91700.
2. Menge, T., et al., *Mesenchymal stem cells regulate blood-brain barrier integrity through TIMP3 release after traumatic brain injury.* Sci Transl Med, 2012. **4**(161): p. 161ra150.
3. Veys, K., et al., *Role of the GLUT1 Glucose Transporter in Postnatal CNS Angiogenesis and Blood-Brain Barrier Integrity.* Circ Res, 2020. **127**(4): p. 466-482.
4. Cheng, Z., et al., *Hydrophilic anti-migraine triptans are substrates for OATP1A2, a transporter expressed at human blood-brain barrier.* Xenobiotica, 2012. **42**(9): p. 880-90.
5. Munji, R.N., et al., *Profiling the mouse brain endothelial transcriptome in health and disease models reveals a core blood-brain barrier dysfunction module.* Nat Neurosci, 2019. **22**(11): p. 1892-1902.
6. Ratelade, J., et al., *Severity of arterial defects in the retina correlates with the burden of intracerebral haemorrhage in COL4A1-related stroke.* J Pathol, 2018. **244**(4): p. 408-420.
7. Ross, E.A., et al., *Mouse stem cells seeded into decellularized rat kidney scaffolds endothelialize and remodel basement membranes.* Organogenesis, 2012. **8**(2): p. 49-55.
8. Holm Nielsen, S., et al., *A novel biomarker of laminin turnover is associated with disease progression and mortality in chronic kidney disease.* PLoS One, 2018. **13**(10): p. e0204239.
9. Beguin, E.P., et al., *Flow-induced Reorganization of Laminin-integrin Networks Within the Endothelial Basement Membrane Uncovered by Proteomics.* Mol Cell Proteomics, 2020. **19**(7): p. 1179-1192.
10. Ishijima, M., et al., *Perlecan modulates VEGF signaling and is essential for vascularization in endochondral bone formation.* Matrix Biol, 2012. **31**(4): p. 234-45.
11. Bader, B.L., et al., *Compound genetic ablation of nidogen 1 and 2 causes basement membrane defects and perinatal lethality in mice.* Mol Cell Biol, 2005. **25**(15): p. 6846-56.
12. Makowska, K.A., et al., *Specific Myosins Control Actin Organization, Cell Morphology, and Migration in Prostate Cancer Cells.* Cell Rep, 2015. **13**(10): p. 2118-25.
13. Lugano, R., et al., *CD93 promotes beta1 integrin activation and fibronectin fibrillogenesis during tumor angiogenesis.* J Clin Invest, 2018. **128**(8): p. 3280-3297.
14. Langenkamp, E., et al., *Elevated expression of the C-type lectin CD93 in the glioblastoma vasculature regulates cytoskeletal rearrangements that enhance*

vessel function and reduce host survival. Cancer Res, 2015. **75**(21): p. 4504-16.

15. Roudnicky, F., et al., *High expression of insulin receptor on tumour-associated blood vessels in invasive bladder cancer predicts poor overall and progression-free survival.* J Pathol, 2017. **242**(2): p. 193-205.
16. Tichet, M., et al., *Tumour-derived SPARC drives vascular permeability and extravasation through endothelial VCAM1 signalling to promote metastasis.* Nat Commun, 2015. **6**: p. 6993.
17. Randhawa, P.K., et al., *The Ras activator RasGRP3 mediates diabetes-induced embryonic defects and affects endothelial cell migration.* Circ Res, 2011. **108**(10): p. 1199-208.
18. Mastrella, G., et al., *Targeting APLN/APLNR Improves Antiangiogenic Efficiency and Blunts Proinvasive Side Effects of VEGFA/VEGFR2 Blockade in Glioblastoma.* Cancer Res, 2019. **79**(9): p. 2298-2313.
19. Miyano, K., et al., *The NADPH oxidase NOX4 promotes the directed migration of endothelial cells by stabilizing vascular endothelial growth factor receptor 2 protein.* J Biol Chem, 2020. **295**(33): p. 11877-11890.
20. Zimmerman, M.W., et al., *Protein-tyrosine phosphatase 4A3 (PTP4A3) promotes vascular endothelial growth factor signaling and enables endothelial cell motility.* J Biol Chem, 2014. **289**(9): p. 5904-13.
21. Tammela, T., et al., *VEGFR-3 controls tip to stalk conversion at vessel fusion sites by reinforcing Notch signalling.* Nat Cell Biol, 2011. **13**(10): p. 1202-13.
22. Murphy, P.A., et al., *Constitutively active Notch4 receptor elicits brain arteriovenous malformations through enlargement of capillary-like vessels.* Proc Natl Acad Sci U S A, 2014. **111**(50): p. 18007-12.
23. Lu, X., et al., *The netrin receptor UNC5B mediates guidance events controlling morphogenesis of the vascular system.* Nature, 2004. **432**(7014): p. 179-86.
24. Goveia, J., et al., *An Integrated Gene Expression Landscape Profiling Approach to Identify Lung Tumor Endothelial Cell Heterogeneity and Angiogenic Candidates.* Cancer Cell, 2020. **37**(1): p. 21-36 e13.
25. Langlois, B., et al., *Vimentin knockout results in increased expression of sub-endothelial basement membrane components and carotid stiffness in mice.* Sci Rep, 2017. **7**(1): p. 11628.
26. Gau, D., et al., *Structure-based virtual screening identifies a small-molecule inhibitor of the profilin 1-actin interaction.* J Biol Chem, 2018. **293**(7): p. 2606-2616.
27. Zhang, H.H., et al., *S-nitrosylation of cofilin-1 mediates estradiol-17beta-stimulated endothelial cytoskeleton remodeling.* Mol Endocrinol, 2015. **29**(3): p. 434-44.
28. Girbl, T., et al., *Distinct Compartmentalization of the Chemokines CXCL1 and CXCL2 and the Atypical Receptor ACKR1 Determine Discrete Stages of*

Neutrophil Diapedesis. Immunity, 2018. **49**(6): p. 1062-1076 e6.

29. Chui, R. and K. Dorovini-Zis, *Regulation of CCL2 and CCL3 expression in human brain endothelial cells by cytokines and lipopolysaccharide.* J Neuroinflammation, 2010. **7**: p. 1.
30. Hauptmann, J., et al., *Interleukin-1 promotes autoimmune neuroinflammation by suppressing endothelial heme oxygenase-1 at the blood-brain barrier.* Acta Neuropathol, 2020. **140**(4): p. 549-567.
31. Minten, C., et al., *DARC shuttles inflammatory chemokines across the blood-brain barrier during autoimmune central nervous system inflammation.* Brain, 2014. **137**(Pt 5): p. 1454-69.
32. Huang, H., et al., *VEGF suppresses T-lymphocyte infiltration in the tumor microenvironment through inhibition of NF-kappaB-induced endothelial activation.* FASEB J, 2015. **29**(1): p. 227-38.
